# Those Record Cards

**Published:** 1921-02-12

**Authors:** 


### Those Record Cards.

At the risk of appearing tedious, we desire to return once again briefly to the subject of medical record cards for panel patients. We have every reason to believe that in spite of the newspaper protests and the complaints made by a considerable body of medical men, the system will be retained in its main particulars, though it is probable that further safeguards will be established and that there may be some simplification of the method, in order to ease the burden on the panel doctor. It is, no doubt, partly the fault of the Ministry of Health that so many misconceptions as to the effect of the scheme have arisen, not only among the general public, but among doctors themselves; and it is significant that in one case in which an official explanation of the system was made in person a hostile body of local medical opinion was in a large measure converted to the Health Ministry's view. The pity is that the panel doctors, as a whole, were not taken into the official confidence before, instead of after, the system was framed and announced.

At the same time, there seems to us to be no solid ground for the chorus of condemnation that has been raised in the Press against every detail of the scheme. It is difficult, either, to understand or to characterise the attitude of a newspaper like *The Times*, which has carried on a ferocious campaign against this plan (as part of its general campaign against the Ministry), and now actually suggests that information acquired by such means as is proposed will prove valueless. "It is not the man who simply records findings or opinions who wins the truth," so runs the sapient argument of *The Times*; "it is the man who proceeds from hypothesis to experiment." It would take up too much space to supply *The Times* with a list of the men who have benefited their fellow-men with discoveries which would have been valueless or incomplete without classified statistics to support them. According to the latest newspaper theory, if it is carried to its logical conclusion, statistical records of any kind are quite worthless. It was in *The Times* that the late Lord Fisher propounded his famous policy of "sacking the lot." He seems to have infected the leader-writers very badly when they are reduced to transferring the principle from naval administration to statistics at large.

